# Chloroform

**Published:** 1848-01

**Authors:** 


### CHLOROFORM.

Among the various articles which have fallen into our hands on the use of the Chloroform, we have thought that which was from the pen of the discoverer—giving, as it does, the mode of its preparation, as well as a general history of its manner of introduction and effects on the system, would be the most proper article for the Register.

For the short time it has been in use, it has been pretty extensively tried by most of the Dentists of our city. Those, indeed, who stood aloof from the use of the Letheon have been among the first to test this new anæsthetic agent. It has appeared to us that some, and we do not specially speak of our city, or of dentists alone, feel particularly delighted that a new agent has made its appearance which enables them to stupify their patients, and at the same time retain their opposition to the Ether. "Consistency thou art a jewel." We have been in the habit, before the Chloroform was discovered, of using the ether, and although always prefering to operate without the exhibition of any agent to allay the pain, yet we have always succeeded quite as well with it as with the Chloroform. We have refused to give ether to some of our patients, and from the closest scrutiny we have been able to make—judging from the effects of both on the system—we should very much hesitate to use the latter where we have refused to use the former. We should refuse to administer either to patients subject to apoplexy, to insanity, to hemorrhage of the lungs, or who labor under any structural derangement of the heart. The use of a proper inhaler we consider more necessary in the use of Chloroform than Ether—a clean handkerchief for the latter answers as well as the best inhalers we have seen, and is generally far more acceptable to the patient—while the former, if suffered to come in contact with the lips of the patient, produces severe pain and blisters. We use, for the administration of the Chloroform, the mouth-piece of Morton's inhaler—filling the brass portion with sponge, which can be removed at pleasure.—Cin. Ed.

